# Barriers and enablers to scholarship for post basic nursing students in clinical service

**DOI:** 10.4102/curationis.v46i1.2385

**Published:** 2023-06-22

**Authors:** Jean-Paul Almaze, Waheedha Emmamally, Petra Brysiewicz

**Affiliations:** 1 School of Nursing and Public Health, College of Health Sciences, University of KwaZulu-Natal, Durban, South Africa

**Keywords:** clinical scholarship, scholarship, clinical nurse, student nurse, clinical setting

## Abstract

**Background:**

Clinical scholarship is defined as an approach that enables evidence-based nursing and the development of best practices to meet the needs of clients efficiently and effectively. However, there are many barriers that impede its progress.

**Objective:**

This study aimed to identify the barriers and enablers to scholarship for post basic nursing students in clinical service areas.

**Method:**

This multimethods study used a structured questionnaire followed by semi-structured individual interviews of post basic nursing students and their lecturers (nurse educators).

**Results:**

The 81 students who completed the questionnaire indicated a lack of support or funding assistance and mentoring, as well as no mechanisms to reward or recognise scholarship as top barriers to clinical scholarship. Top enablers were noticed as reward mechanisms in place, more protected time and availability of role models and mentoring. Twelve respondents engaged in the qualitative phase and three categories emerged from the data, namely (1) resource dependent, (2) ‘what’s the use of research’, (3) making a change.

**Conclusion:**

It has been shown that there is a need to adopt and promote a culture of clinical scholarship to ensure that the best available evidence is being utilised by nurses to effectively manage their patients; however, to support clinical scholarship, resources are needed.

**Contribution:**

This study highlighted the lack of funding and resources as being a major barrier to scholarship, together with an institutional culture that did not promote clinical scholarship. Providing protected time, mentoring and criteria for promotion and reward based on scholarship is viewed as enabling.

## Introduction

Clinical scholarship is defined as an approach that enables evidence-based nursing and the development of best practices to meet the needs of clients efficiently and effectively (Wilkes, Mannix & Jackson 2013, Zullig, Deschodt & De Geest 2019). Although among nurses, the definition of clinical scholarship is not well understood (Carter et al. 2017; Wilkes et al. 2013), efforts should be made to encourage them to engage in scholarship thereby improving patient care and, ultimately, patient outcomes (O’Connor & Peters 2014; Roets, Botma & Grobler 2016; Weston & Hudson 2014; Wilkes et al. 2013).

Clinical scholarship should not only be viewed as clinical research or clinical proficiency but also as a check point that seeks to address pertinent questions about patient care. The hallmark of clinical scholarship questions traditional notions of nursing, which may then be systematically challenged so that improved patient care outcomes may be forecasted (Sigma Theta Tau International Clinical Scholarship Task Force 1999; Sigma Theta Tau International 2015). Weston and Hudson (2014) agreed that clinical scholarship should be the ‘nucleus’ of nursing practice, creating opportunities for learning, leading to improved clinical practice and care enhancement. Clinical scholarship embraces knowledge from other disciplines to expand understanding and highlights the need for peer-reviewed documentation, logical presentation and effective dissemination; also creating opportunities for clinical nurses to integrate nursing theories in practice (Papathanasiou, Tsaras & Sarafis 2014). Weston and Hudson (2014) emphasise that clinical scholarship is not just about solving immediate issues related to illnesses but it also explores how nurses can improve clinical practice on a broader scale.

Nurses working in the clinical service areas should be encouraged to appreciate and embrace clinical scholarship as an essential element of the nursing profession to guide and give answers to important issues in the clinical arena and practice (Carter et al. 2017; Weston & Hudson 2014). Clinical scholarship is an approach that achieves positive patient outcomes (Makic et al. 2013).

Despite this, the application and promotion of clinical scholarship in the clinical arena has been limited. Although recently efforts are underway in some countries to promote clinical scholarship, clinical nurses globally have reported on the numerous individual and organisational obstacles (such as a lack of research knowledge, skills, time etc.) to conducting research in the clinical areas thus making clinical scholarship difficult to conceptualise and implement (Carter et al. 2020; Wilkes et al. 2013). A need to focus on clinical specialist nurses in this regard has emerged as they are perfectly positioned between the clinical and academic worlds and best situated to be asking questions to address important clinical problems (Oster, Ludwigson & Lewis 2020) and are thus able to provide insight into barriers and enablers to clinical scholarship.

## Aim of the study

The aim of this study was to identify the barriers and enablers to clinical scholarship for post basic nursing students in clinical service areas.

## Methods

This multimethods study (Anguera et al. 2018) comprised a non-experimental, quantitative descriptive survey as well as individual semi-structured interviews with students and lecturers. Boyer’s Framework of Scholarship (Boyer 1990) was used to guide the study as it proposes that a scholar engages in four essential and interrelated areas of scholarship in the pursuit of knowledge. These are scholarship of discovery (searching for problems and explanation of research); scholarship of integration (interpreting the findings of the research and sharing with and across the discipline); scholarship of teaching (creating interaction between the one delivering the knowledge and the one receiving the knowledge) and scholarship of application (translating the knowledge so that it impacts positively on society). Smith et al. (2012) agree that incorporating these four components could assist nurses to engage in the activities of clinical scholarship.

### Research setting and respondents

The research was conducted at a selected university offering a wide variety of nursing programmes located in KwaZulu-Natal, South Africa.

The study targeted all registered post basic (post registration) nursing students and their lecturers (nurse educators) involved in the following undergraduate (a comprehensive practice degree for post registration nurses) or postgraduate clinical programmes, for example, Critical Care and Trauma (*n* = 46), Oncology and Palliative Care (*n* = 21), Advanced Midwifery and Neonatal Nursing (*n* = 39) and Nursing Management (*n* = 56). Following advice from a statistician and considering the small population size, a census sampling strategy was identified and all eligible respondents (*n* = 162) were invited to participate in the study.

### Research tool

Guided by Boyer’s framework (1990), the researchers developed a questionnaire adapted from a previous study by Smesny et al. (2007).

The questionnaire was made up of the following sections: (1) demographics (7 questions), (2) barriers to clinical scholarship (13 questions) and (3) solutions or enablers to these barriers (16 questions). The tool asked the respondents to rate their responses on a 4-point Likert scale ranging from 1 = strongly disagree, 2 = disagree, 3 = agree, and 4 = strongly agree. Owing to minimal responses in some categories, the 4-point Likert scale was condensed to form a 3-point Likert scale where responses of ‘strongly disagree’ and ‘disagree’ as well as ‘agree’ and ‘strongly agree’ were grouped together to improve stability and generalisability (Boone & Boone 2012).

The interview guide for the qualitative interviews was developed using Boyer’s framework (1990) and asked respondents to describe barriers and facilitators to the different components of scholarship, as well as responses from the questionnaire, which needed additional probing.

### Data collection process

Data collection took place after ethical clearance and permission was obtained from the university research ethics committee as well as the registrar of the university and head of nursing. In collecting the quantitative data, the researcher (J.-P.A.), a postgraduate student himself not registered in any of the programmes involved in the study organised to meet with the lecturers of the respective programmes to arrange a convenient time and venue to meet the nursing students. Data were collected during the students break time or after class and the survey took approximately 15–20 min to complete. Questionnaires were distributed to the respondents after obtaining written informed consent and all questionnaires were distributed and collected on completion by the researcher (J.-P.A.)

The individual semi-structured interviews were held with eight students and four lecturers who indicated their willingness to be interviewed. These interviews asked the respondents about barriers to clinical scholarship and to also suggest ways to promote clinical scholarship in the clinical service area. The respondents were contacted individually and a convenient time was arranged. The interviews were conducted in a quiet venue on campus and were audio recorded with the respondent’s permission. The researcher (J.-P.A.) faced unexpected challenges during this phase as university strike action meant that respondents were not available on campus and thus interviews were delayed by a few weeks. The decision that data redundancy had been reached was decided on by the research team through extensive discussion.

### Data analysis

Using the Statistical Package for the Social Sciences (SPSS), version 23, and with the assistance of a statistician, descriptive statistics consisting of frequencies and percentages were carried out. The analysis of the qualitative data was performed manually using qualitative content analysis (Graneheim & Lundman 2004, Erlingsson & Brysiewicz 2017) where the data collected were collected and transcribed verbatim by the researcher (J.-P.A.), thereafter coding and development of categories was undertaken by the whole research team (the student and his two research supervisors).

### Rigor

A pilot study of the questionnaire, involving nursing students (five) from the sample, showed no difficulty in answering the tool, thus no changes were made and their data were not included in the final analysis. Cronbach’s alpha was calculated at 0.94, indicating excellent internal consistency (Clark & Creswell 2015). Face validity was achieved through consultation with two nursing clinical and research experts from the research setting and no changes were made.

For the qualitative data, the research team attempted to make the transcripts of interview materials (the ‘respondent voices’) available in sufficient detail in the text to provide the reader with an opportunity to follow the researchers’ move from data collection to data interpretation. Data analysis was carried out independently by the researcher (J.-P.A.) and then discussed and interrogated in detail by the research supervisors (W.E. & P.B.), who are experienced qualitative researchers. The researchers also attempted to provide sufficient detail of the research process (Lincoln & Guba 1985) and the findings were validated by the respondents.

### Ethical considerations

Ethical clearance to conduct this study was obtained from the University of KwaZulu-Natal Humanities and Social Sciences Research Ethics Committee (No. HSS/1550/016M) and gatekeeper permission from the university registrar and head of nursing was obtained. Respondents were made aware of the purpose of the research and that they are under no obligation to participate in the research, and that refusal would not affect their studies in any way. All data collected from respondents were de-identified and stored securely in a locked cupboard and password protected computer; only accessible to the research team. Data were stored with the research supervisors for a period of 5 years.

## Results

### Quantitative survey

A total of 81 nursing students completed the questionnaire indicating a response rate of 50%.

#### Demographic data

There were 81 nursing students (only 2 males) who ranged in age from 20 to over 41 years old. Forty-five of the respondents had between 0 and 10 years of experience in nursing and 71 were post registration undergraduate nursing students and 10 were postgraduate students (see Table 1).

**TABLE 1:** Demographic data – quantitative data (*N* = 81).

Variable	Respondents	
	*n*	%
**Gender**		
Male	2	2.5
Female	79	97.5
**Age**		
20–25	3	3.7
26–30	15	18.5
31–35	19	23.5
36–40	16	19.8
41 >	28	34.6
**Years of experience in nursing**		
0–10	45	55.5
11–20	23	28.4
21–30	10	12.3
31–40	3	3.7
**Years of experience in clinical specialty**		
0–10	49	60.5
11–20	7	8.6
No experience	17	21.0
No response	8	9.9

#### Barriers to clinical scholarship

The top-rated barriers for clinical scholarship that were report and need to be considered were; *lack of support or funding mechanisms to support scholarship of application or teaching in funding agencies or organisations* (*n* = 64, 84.0%), *clinicians need assistance or mentoring in writing publications or other mentoring activities related to scholarship* (*n* = 67, 82.7%) and *no mechanisms to reward or recognise scholarship of teaching or scholarship of application locally or nationally* (*n* = 64, 79.0%). The lowest rated barrier was: *clinical services requirements and teaching reduce opportunities for scholarship* (*n* = 47, 58.0%) (see Table 2).

**TABLE 2:** Barriers and enablers to clinical scholarship.

Barriers to clinical scholarship	Disagree		Agree		Enablers to clinical scholarship	Disagree		Agree	
	*n*	%	*n*	%		*n*	%	*n*	%
A lack of support or funding mechanisms to support scholarship of application or teaching in funding agencies or organisations	13	16.0	68	84.0	Re-examine criteria for promotion of clinical faculty and create a structural framework within the school or college as well as the institution to foster, assess, and reward all types of scholarship.	6	7.4	75	92.6
Clinicians need assistance or mentoring in writing publications or other mentoring activities related to scholarship	14	17.3	67	82.7	Provide more protected time and/or uninterrupted time and resources to perform scholarship of all types	7	8.6	74	91.4
No mechanisms to reward or recognise faculty scholarship of teaching or scholarship of application locally or nationally	17	21.0	64	79.0	Using senior faculty role models, create a collaborative mentoring programme, which may include training on how to approach writing articles and grantsmanship.	7	8.6	74	91.4
Health-student debt load or salary is too low leading to a lack of interest in positions requiring scholarly activities	18	22.2	63	77.8	Create synergy between research and practice	8	9.8	73	90.1
Few role models or mentors for scholarship and clinical activities	19	23.5	62	76.5	Assign more importance to the special contributions of clinician educators and use a variety of methods to assess their abilities	9	11.1	72	88.9
Discipline members are unaware of other forms of scholarship as it relates to promotion and tenure	20	24.7	61	75.3	Include a similar reward system for all forms of scholarship and educate clinicians and administrators on the different forms of scholarship	10	12.4	71	87.6
Difficulty in becoming a competent clinician who can keep up with complexity of sciences	23	28.4	58	71.7	Develop new faculty positions to foster various types of scholarship and clinical practice	10	12.4	71	87.6
Work of clinician educator is less amenable to publication or to presenting their scholarship or activities	24	29.7	57	70.3	Encourage interdisciplinary cooperation and create cross disciplinary initiatives to link the physician scientist and/or basic researcher to the clinician	10	12.4	71	87.6
Promotion and tenure guidelines are not consistent with clinical practice faculty job specification	27	33.4	54	66.7	Create a clinician-educator researcher by providing training in master’s levels or PhD in the area of education	11	3.6	70	86.4
Institutional culture does not foster or promote scholarship	28	34.6	53	65.4	Develop and implement a two-track system (clinical track and research track) with an emphasis on practice	11	13.6	70	86.4
A lack of interdisciplinary cooperation between clinicians and academic clinical scholarship lack of collegiality	28	34.6	53	65.4	Regularly review balance of activities in academic posts, particularly between service work, teaching and research	11	13.6	70	86.4
Time frame for tenure and promotion related to development and demonstration of scholarship of application, teaching, etc. may be longer than current time frames	31	38.3	50	61.7	Develop criteria for recognising and rewarding faculty scholarship related to service including clinical activities, community service, public health service practice, and professional organisation activities	12	14.8	69	85.2
Clinical services requirements and teaching reduce opportunities for scholarship	34	42.0	47	58.0	Develop a thematic based faculty development curriculum to catalyse clinician faculty to become involved in scholarly projects that increase enthusiasm for research	12	14.8	69	85.2
					Create a model of scholarship that requires a high level of discipline-related expertise, breaks new ground or is innovative, can be replicated, documented, peer-reviewed, and has a significant impact	13	16.0	68	83.9
					Design post-graduate residencies to be geared more towards research rather than education and establish more research training fellowships	14	17.8	67	82.2
					Using Boyer’s model of scholarship, requires faculty to work in four areas of scholarship including the areas of discovery, teaching, integration and application	15	18.5	66	81.5

#### Enablers to clinical scholarship

The top enablers to clinical scholarship were: *re-examined criteria for promotion and reward on scholarship* (*n* = 75, 92.6%), *provide more protected time and/or uninterrupted time and resources to perform scholarship of all types* (*n* = 74, 91.4%) and *using lecturer role models, create a collaborative mentoring programme which may include training on how to approach writing papers and grantsmanship* (*n* = 74, 91.4%). The item with the lowest score was using *Boyer’s Model of Scholarship* (*n* = 66, 81.5%).

#### Qualitative individual interviews

The 12 individual semi-structured interviews were held with eight students and four lecturers (respondent code; NS = nursing student and CE = clinical expert/lecturer, age, years of experience).

There were three categories that emerged from the data regarding the barriers and enablers to clinical scholarship and these included; resource dependent, ‘what’s the use of research’ and making a change.

### Resource dependent

Respondents indicated that a major barrier to their own clinical scholarship development was the fact that it was very dependent on having appropriate resources. For many nurses these resources were not available to them. A respondent explained:

‘… problem of not been motivated to [*pursue further*] study and involved in clinical scholarship … is the lack of money which most of the time is the case … sometime they [*clinical nurses*] just cannot afford to further their studies due to lack [*of*] or no funding …’ (NS 8, 36–40 years old, 5 years experience)

An important resource identified was experts to assist:

‘resources that could be a barrier will be … if we don’t have expert[*s*], people who are knowledgeable about clinical scholarship that would help transfer the knowledge about clinical scholarship.’ (NS 4, > 41 years old, 20 years experience)

‘the institution need[*s*] to make sufficient fund[***s***] available for individual[*s*] who are interested in conducting [*a*] small project that will benefit and contribute towards the development of clinical scholarship.’ (NS 4, > 41 years old, 20 years experience)

A respondent said:

‘I think people involving in clinical scholarship development, should be supported by other colleagues and rewarded … I think this could encourage and gear up people more towards the development and sustain of clinical scholarship.’ (NS 2, 31–35 years old, 8 years experience).

The enablers or solutions suggested was that the institutions need to make such resources available and incentivise the staff to conduct research and pursue various scholarship activities. A respondent said:

‘I think people involving in clinical scholarship development, should be supported by other colleagues and rewarded … I think this could encourage and gear up people more towards the development and sustain[*ability*] of clinical scholarship.’ (NS 2, 31–35 years old, 8 years experience)

‘What’s the use of research?’

The second category that emerged as a barrier to clinical scholarship described how some nurses do not see the relevance and importance of research and its value in clinical practice at the bedside, as one respondent, a nurse educator, explained:

‘They [*nurses*] are out there making money and research was nice to know … have to do it for the university and complete the study… sometime[*s*] they don’t see what is the use of research.’ (CE 2, > 41 years old, 36 years experience)

A respondent agreed and went further to say:

‘… people don’t think that research findings are possible to implement in [*the*] clinical area.’ (CE 3, > 41 years old, 39 years experience)

Another respondent, a nurse educator, also emphasised the reluctance expressed by nursing students:

‘… students are tired and they just want to get over and done with it [*research*] [*in*] practice mostly all the students are hesitance of research, they worry about it, [*that*] there so much work to be done …’ (CE 2, > 41 years old, 36 years experience)

In order to try to address this problem the respondents said:

‘Nurses should be involved in research, we should not think it is only for researcher[*s*] or only when you are doing [*your*] master[*s*] or doctorate.’ (NS 2, 31-35 years old, 8 years experience)

### Making a change

In order to enable and promote clinical scholarship, the respondents described how it is important to foster a change in the way in which nurses view research and clinical scholarship activities. There are however difficulties with this as one respondent explained:

‘Some [*nurses*] don’t feel comfortable with changing, because they like doing their comfort thing, they are comfortable in their old attitude and ways of doing things, which they developed their routine that they don’t want to change …’ (NS 3, > 41 years old, 10 years experience)

One respondent admitted that another problem for nurses in developing clinical scholarship is that they are not confident and assertive enough to defend their own research:

‘We [*nurses*] are not assertive enough to tell the doctor, no, no, no … that [*my*] study … also is worthy [*that*] this is what it say[*s*].’ (CE 3, > 41 years old, 39 years experience)

In considering solutions to this problem a respondent explained:

‘I think the division between education and clinical practice is one important thing that must be avoided. That distance between clinical and the theory of college or university, that distance must be narrow.’ (CE 3, > 41 years old, 39 years experience)

## Discussion

The quantitative data indicated that the top-rated barriers to clinical scholarship included the lack of support or funding mechanisms, the need for assistance or mentoring in various activities related to scholarship and no mechanisms currently available to reward or recognise scholarship. The top enablers cited were to institute criteria for promotion and reward regarding clinical scholarship, provide more protected and/or uninterrupted time and resources to perform scholarship, as well as to provide experts and mentors to guide and assist in various scholarship activities.

The qualitative data revealed that the respondents viewed a major barrier to their own clinical scholarship development being the lack of appropriate resources such as money for studies and research as well as having access to people with expertise to guide them in the process of evaluating their nursing care in order to institute improvements where necessary. An additional barrier to clinical scholarship was that nurses struggle to see the relevance and importance of research and its value in clinical practice. The respondents went further to suggest that in order to enable clinical scholarship, it is important to foster a change in the way in which nurses view research and clinical scholarship activities.

From the study’s finding, it has been highlighted that a lack of funding and resources were identified as being a major barrier to scholarship in limiting the nurses’ involvement in scholarly activity and Anderson et al. (2013) agree that this is often a major setback. In an Australian study, more than 90% of the respondents were unable to pursue further educational training towards scholarship development because of a lack of financial aid (O’Connor & Peters 2014). Pintz et al. (2018) suggest that it is necessary to establish and/or sustain a designated budget with funds to recognise, support and reward nursing research activities and accomplishments.

The respondents of this study claimed that often the institutional culture does not promote clinical scholarship. In order to keep the nursing profession moving forward and improving patient outcomes, the Sigma Theta Tau Clinical Scholarship Task Force in 1999 recommended that the healthcare professional must take the responsibility to have a vision based on a culture that supports clinical scholarship (STTI Clinical Scholarship Task Force 1999). In order to support this however Whittaker, Kernohan and McLaughlin (2014) suggest that participation in research activity is important and that organisations should create the opportunity for staff development so that the individual can be made to see their ability. Pintz et al. (2018) agreed and supported the need of establishing a culture of scientific enquiry within organisations. In answering this, some countries are developing national frameworks and/or structures to support and develop clinical researchers and creating clinical academic positions (Health Education England/National Institute for Health Research 2017). Weston and Hudson (2014) state that in order for clinical scholarship to gain support, the clinical arena should value the generation of knowledge aimed at enhancing clinical practice.

Among other barriers, the respondents stated that clinical nurses need assistance in various research or scientific tasks, for example, how to write an article and to appreciate that clinical scholarship does not reach its ultimate value until it is shared. This was also identified by O’Connor (2019) where findings from their study suggested that clinical scholarship be integrated in the curriculum activity to better equip the nurses. In support of this, Roets et al. (2016) suggest that nurses with a baccalaureate degree may be better equipped to go beyond the development of research and skills and that this may help the clinical nurse specialist to generate knowledge in clinical practice and build theory. Blanchard, Visintainer and La Rochelle (2015) and Siedlecki and Albert (2017) state that having a good role model or mentor is needed to assist in providing important guidance in helping clinical nurses to integrate research and practice. Carter et al. (2020) suggests that partnerships between practice and academic institutions may be a way forward to build and support research capacity in clinical nurses such as a joint nurse scientist role, which then fosters a partnership between academia and service.

Siedlecki and Albert (2020) do however caution that removal of the barriers does not guarantee an increase in research activities; clarity regarding the understanding of clinical scholarship is needed and attention should be a given to ensuring that the elements and qualities of a clinical scholar is clear to nurses. Casamitjana et al. (2022) also suggest that in order to achieve scientific equity in health research, gaps in individual and institutional capacity and infrastructure in low- and middle-income countries must be addressed.

### Limitations of the study

A major limitation was the sample size that affects the generalisability of the study, however the researchers attempted to overcome this through the use of multiple methods. The second limitation identified was that the study included only one setting, thus different areas may be experiencing different challenges. The developed research tool used in the quantitative aspect of the study needs further validation and wider use.

### Recommendations

It is recommended that a study with a larger sample size and incorporating different research settings should be conducted to enable generalisability of the findings. Current nursing curricula should be interrogated to ensure that they are providing sufficient understanding of clinical scholarship, its essential role within the nursing profession and ways to support it within the clinical service arena. The clinical settings should work towards creating a conducive environment for clinical scholarship development and there should be more emphasis placed on the importance of integrating research into clinical practice, as well as research utilisation in the clinical arena.

## Conclusion

This study has highlighted the barriers and enablers towards clinical scholarship. Nurses are the most dominant cadres in a healthcare system and are key role players in providing patient care. For this reason, nurses need to embrace and promote a culture of clinical scholarship, thus ensuring best available evidence is being utilised to best manage their patients, however resources are needed in order to make this a reality.
